# Planting of neonicotinoid-coated corn raises honey bee mortality and sets back colony development

**DOI:** 10.7717/peerj.3670

**Published:** 2017-08-14

**Authors:** Olivier Samson-Robert, Geneviève Labrie, Madeleine Chagnon, Valérie Fournier

**Affiliations:** 1 Centre de recherche en innovation sur les végétaux, Université Laval, Québec, Canada; 2 Centre de recherche sur les grains Inc., Saint-Mathieu-de-Beloeil, Québec, Canada; 3 Département des Sciences Biologiques, Université du Québec à Montreal, Montréal, Québec, Canada

**Keywords:** *Apis mellifera*, Clothianidin, Insecticide, Pollinator, Seed treatment, Thiamethoxam, Honey bee, Intoxication, Population dynamic model, Colony health

## Abstract

Worldwide occurrences of honey bee colony losses have raised concerns about bee health and the sustainability of pollination-dependent crops. While multiple causal factors have been identified, seed coating with insecticides of the neonicotinoid family has been the focus of much discussion and research. Nonetheless, few studies have investigated the impacts of these insecticides under field conditions or in commercial beekeeping operations. Given that corn-seed coating constitutes the largest single use of neonicotinoid, our study compared honey bee mortality from commercial apiaries located in two different agricultural settings, i.e. corn-dominated areas and corn-free environments, during the corn planting season. Data was collected in 2012 and 2013 from 26 bee yards. Dead honey bees from five hives in each apiary were counted and collected, and samples were analyzed using a multi-residue LC-MS/MS method. Long-term effects on colony development were simulated based on a honey bee population dynamic model. Mortality survey showed that colonies located in a corn-dominated area had daily mortality counts 3.51 times those of colonies from corn crop-free sites. Chemical analyses revealed that honey bees were exposed to various agricultural pesticides during the corn planting season, but were primarily subjected to neonicotinoid compounds (54% of analysed samples contained clothianidin, and 31% contained both clothianidin and thiamethoxam). Performance development simulations performed on hive populations’ show that increased mortality during the corn planting season sets back colony development and bears contributions to collapse risk but, most of all, reduces the effectiveness and value of colonies for pollination services. Our results also have implications for the numerous large-scale and worldwide-cultivated crops that currently rely on pre-emptive use of neonicotinoid seed treatments.

## Introduction

In recent years, decline in pollinators, both wild and managed, has garnered much attention, prompting considerable amount of research (see Lundin et al., 2015; Godfray et al., 2015 for review). In light of theses studies, a suite of numerous and interacting factors have been highlighted, including loss of foraging resources due to habitat loss and homogenization, introduction of invasive species, climate change, parasites, pathogens, loss of genetic diversity, beekeeping practices, and exposure to pesticides (Potts et al., 2010; Vanbergen et al., 2013; Goulson et al., 2015; Gill et al., 2016; Klein et al., 2017). The role of pesticides, especially those of the neonicotinoid family, have been of particular concern (Tennekes & Sánchez-Bayo, 2011). While a growing body of evidence has shed light on the multiple routes of exposure to these insecticides and their negative impacts of bee health (Maini, Medrzycki & Porrini, 2010; Pisa et al., 2015; Samson-Robert et al., 2015; Lundin et al., 2015; Tsvetkov et al., 2017; Woodcock et al., 2017) their implications in colony decline are still much debated (Blacquière & Van der Steen, 2017). Current gaps in our knowledge, particularly at colony-level (Godfray et al., 2015) and from field-based approaches (Lundin et al., 2015), contribute to uncertainty surrounding these insecticides.

In the province of Quebec alone, prophylactic neonicotinoid seed treatment in corn and soybean represents about 1.5 million acres of land every year (ISQ, 2016), largely located in the southern area of the province. This area is also home to a third of the province’s commercial beekeepers, with more than 12,000 colonies (ISQ, 2015). The use of neonicotinoid as seed treatments has diversified and multiplied the routes by which honey bees and other pollinators may be contaminated, consequently increasing the risk of exposure to these systemic insecticides. It is now known that planting of seeds coated with neonicotinoid releases particulate matter contaminated with the insecticide into the environment (Greatti et al., 2003; Greatti et al., 2006; Girolami et al., 2012; Krupke et al., 2012; Sgolastra et al., 2012; Pistorius et al., 2015), and honey bees in agricultural areas are likely to come into contact with clouds of contaminated dust while foraging (Goulson, 2013; Godfray et al., 2015). Airborne particulate matter is also highly susceptible to drifting, settling and thereby contaminating flowers, which increases the risk of intoxication when honey bees collect pollen or nectar from contaminated vegetation (Goulson, 2013; Godfray et al., 2015; Long & Krupke, 2016). It has also been establish that only a fraction of the seed coating’s active ingredient (1.6–20%) can be absorbed by the crop, thus allowing for the bulk of the remaining insecticide to stay in the soil, leach down and reach surface waters (Sur & Stork, 2003; Goulson, 2013). Ubiquitous contamination of surface water with neonicotinoid insecticides combined with important water requirements for colony developement in the spring has also been higlighted as harmful for honey bee health (Samson-Robert et al., 2014; Chrétien et al., 2017). In the province of Quebec and Ontario (eastern Canada), the planting period of treated corn has repeatedly been associated with reported honey bee deaths (PMRA, 2017).

Given the extent to which neonicotinoid seed coating is used and its particular proximity with beekeeping operations, we initiated this work to establish whether large-scale use of treated corn was harmful to honey bee colonies and the beekeeping industry. We conducted a 2-year study using corn fields from commercial growers and commercial apiaries, therefore insuring the most realistic conditions. First, we monitored colony mortality during the corn planting period and analysed dead honey bees for pesticide residues. Second, we used a colony population dynamic model to assess the extent to which honey bee mortality might disrupt colony growth and estimate its resulting impacts on the Southern Quebec’s beekeeping industry.

## Materials and Methods

### Experimental setting

**Figure 1:**
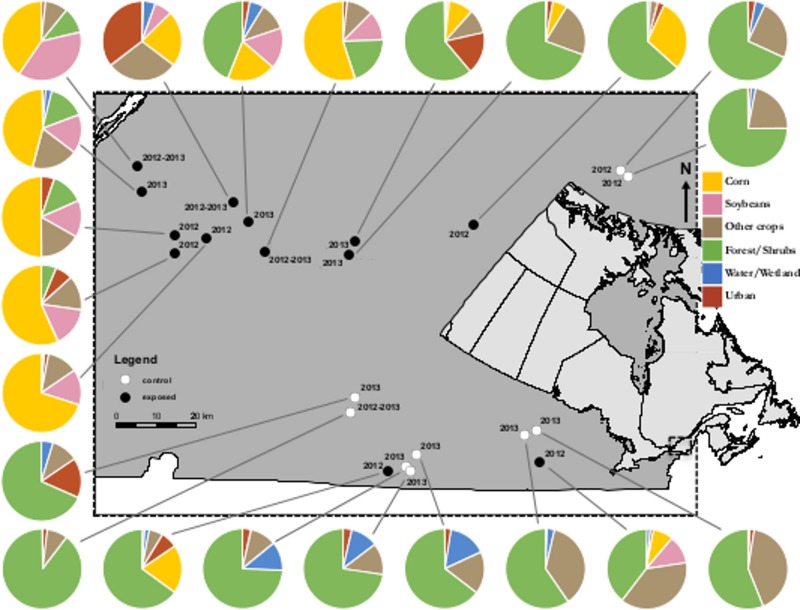
Map of study sites. Study area in Southern Quebec and locations of commercial apiaries in corn-free areas (open circles) or corn-dominated areas (filled circles). Circle charts describe land cover of each site within a 3 km radius.

Field trials took place in the southern part of Quebec, in Eastern Canada in a region with a historically high level of land use for agricultural purposes. This area is Quebec’s most productive agricultural region and produces over 60% of the province’s corn and soybean crops. Study sites (apiaries) were selected among seasonal commercial bee yards customarily and historically managed by beekeepers. Exposed apiaries (*n* = 9 for 2012, *n* = 7 for 2013) were located within a 500 m radius from a treated-corn field, within a broader landscape (3 km radius) dominated by neonicotinoid-treated corn fields (exposed treatment). Control apiaries (*n* = 3 for 2012, *n* = 7 for 2013) were located in an area where the main crops are hay, wheat, oats, barley and rye and with minimal distance of 3 km from any crop planted with neonicotinoid seed coating. Minimal distance between same treatment sites was 3 km and 17 km in between exposed-control sites (Fig. 1).

### Honey bee colonies

Commercial honey bee hives were placed at every study site by their respective beekeepers/owners according to the usual seasonal timing of such procedures, either before, or at the very beginning, of large-scale corn planting operations. All colonies remained on site for the duration of the experiment and were then moved for pollination purposes by owners. All study sites were factual customary bee yards locations and colonies were entirely managed by owners. However, colony management was standardised between beekeepers: all colonies (*Apis mellifera* hybrid Italian stock) were reared in Langstroth hives, treated with oxalic acid for varroa mite control and supplied with *ad libitum* pollen supplements. In each apiary, five colonies were randomly selected from among those with similar colony strength at the beginning of the experiment (five frames of bees and four frames of brood). In 2012 and 2013, four beekeepers managed colonies exclusively in exposed apiaries and two beekeepers managed colonies in both exposed and controled apiaries, for a total of six beekeepers per year.

### Mortality index

A one square meter (1 m^2^) piece of pale beige cloth was placed in front of each experimental hive. Every 48 h, dead honey bees found on the cloth were counted to evaluate mortality levels and then removed. In 2012, the sampling period went from May 3rd to June 15th; in 2013, from May 6th to May 24th. By definition, mortality rate is the measure of the precise number of deaths in a known population, per unit of time. Although colony strength can be estimated, the exact number of living bees it comprises cannot be known, especially in daytime when foragers are out of the hive. Similarly, the number of dead bees cannot be established precisely since honey bees exhibit necrophoric behaviour to limit the spread of disease and parasites and to avoid attracting predators (Visscher, 1983). Furthermore, estimating mortality based on the number of dead bees in front of the hive does not take into account the numerous deaths that might occur away from the hive, for example, when bees die in the field after a lethal exposure to pesticides or when they become disoriented and fail to return to the hive (Porrini et al., 2002). In fact, most of the mortality happens away from the colony and dead bees found in front of the hive only account for 1 to 20% of all deaths (Johansen & Mayer, 1990). As such, the estimate of mortality is referred to subsequently as a “mortality index” and acts as the best proxy for the comparison of mortality rates, while still vastly undervaluing honey bee losses. When mortality exceeded 100 dead bees per colony (minimum amount required for chemical analyses), specimens were collected for ulterior residue analyses.

### Chemical analysis

Dead honey bee samples were analyzed using a modified version of the QuEChERS method originally described by Anastassiades et al. (2003). Briefly, 15 ml of acetonitrile was added to 10 mg of a previously ground and homogenized sample of honey bee bodies. Extract was shaken vigorously for 1 min using a Vortex mixer at maximum speed. Afterwards, 0.6 mg of anhydrous magnesium sulfate and 1.5 mg of anhydrous sodium acetate were added to the extract, which was then vortexed for 1 min and centrifuged for 10 min at 3,450 rpm. An aliquot of 1 ml was transferred to a glass vial, and 60 µl of methanol and 20 µl of isoprocarb standard solution (10 ppm) were added. The solution was then strained through a 0.45 µm PTFE filter and 10 µl were analyzed by liquid chromatography/mass spectrometry utilizing a Waters Acquity LC interfaced to a Waters Xevo TQ MS (Halo C-18 columns, 4.6 solid core × 50 mm porous outer shell with 2.7 µm particle). The mass spectrometer was positioned in a positive electrospray mode and utilized a different MS/MS scan for each of the pesticides monitored. Liquid chromatography injections were repeated three times. Parent pesticides and metabolites were identified based on comparisons of their chromatographic retention time with known standards and mass abundance ratios to at least two fragment transitions. The ion ratios between the two transitions had to comply with a maximum difference of 20% with the calibration standard. This multi-residue method allows for detection of over 400 agrochemical compounds at parts per billion concentration levels. Analyses were performed by a team of professional chemists from the Laboratory of food analyses at the Quebec Ministry of Agriculture (MAPAQ).

### Intracolonial population dynamic model

The commercial beekeeping context in which this study was carried did not allow for controlled conditions after corn planting as colonies were immediately moved for pollination services of different crops (e.g., lowbush blueberry, cranberry), under different climates, at varying distances from our observation locations and for different durations. Therefore, population modeling was used to simulate colony dynamic. The model is based on the mathematical models of Khoury, Myerscough & Barron (2011) and operates under different scenarios based on the initial size of the colony’s population, queen’s egg-laying rate and mortality levels while only considering the population of female worker bees. In all simulations, the evolution of a typical colony was considered during the first three months of the beekeeping seasons and encompasses the corn planting period in our area (roughly the whole month of May) and the blooming period of the two most important pollinator-dependant crops. The simulation-modelling period starts when colonies emerge from their wintering confine, around mid-April. Population size is usually between 10,000 and 14,000 female worker bees, i.e., the average colony size at the beginning of the season in our study area. Forager bees were set to account for 25% of the colony’s population (Fahrbach & Robinson, 1996). The daily egg-laying rates were set to three realistic levels, namely a rate allowing for fast (1,750 eggs/day), typical (1,500 eggs/day) and slow (1,250 eggs/day) colony development (Winston, 1991). Natural forager death rate was set to 0.08 and 0.14 forager bees per day as reported in scientific literature (Rueppell, Kaftanouglu & Page Jr, 2009; Russell, Barron & Harris, 2013; Betti, Wahl & Zamir, 2014). The term *w* reflects the emergence rate from brood to adult and was set to 16,000 to simulate a fast-growing colony typical of spring and early summer (Cresswell & Thompson, 2012). Other dynamic parameters remained unchanged from Khoury, Myerscough & Barron (2011) initial model. Simulations were run under the hypotheses of (1) typical and constant forager death rate with no or insignificant foragers’ exposure, and (2) forager death rate increased by pesticide exposure during a 30-days corn planting period. In the later configuration, exposed foragers death rate was raised by a factor of 3.51 as determined by our mortality survey (see ‘Results’).

### Statistical analyses

The honey bee mortality index data set was analysed using a generalized randomized block design (GRBD) with repeated measures. The experimental units consisted of a group of five colonies located in honey bee yards. GRBD refers to the randomization of study sites within treatments (exposed and control) and within blocks comprised of both study years (2012 and 2013). In this model, treatments and dates were considered as fixed factors, while years and study sites were considered as random factors. Dead honey bees were collected from the same colonies at repeated intervals throughout the corn planting season. Repeated measures design was used to address the longitudinal nature of the data set. The correlation structure for observations within the same experimental unit was selected based on Akaike Information Criterion (AIC). The normality assumption was tested using the Shapiro–Wilk’s statistic, and the homogeneity of variances was verified using the usual residual plots. Box–Cox power transformation was used to remedy violation of the model’s assumptions. Statistical analyses were performed with the lme() function in the nlme package of R software (V 3.0.2) with the significance level set at 0.05.

**Figure 2:**
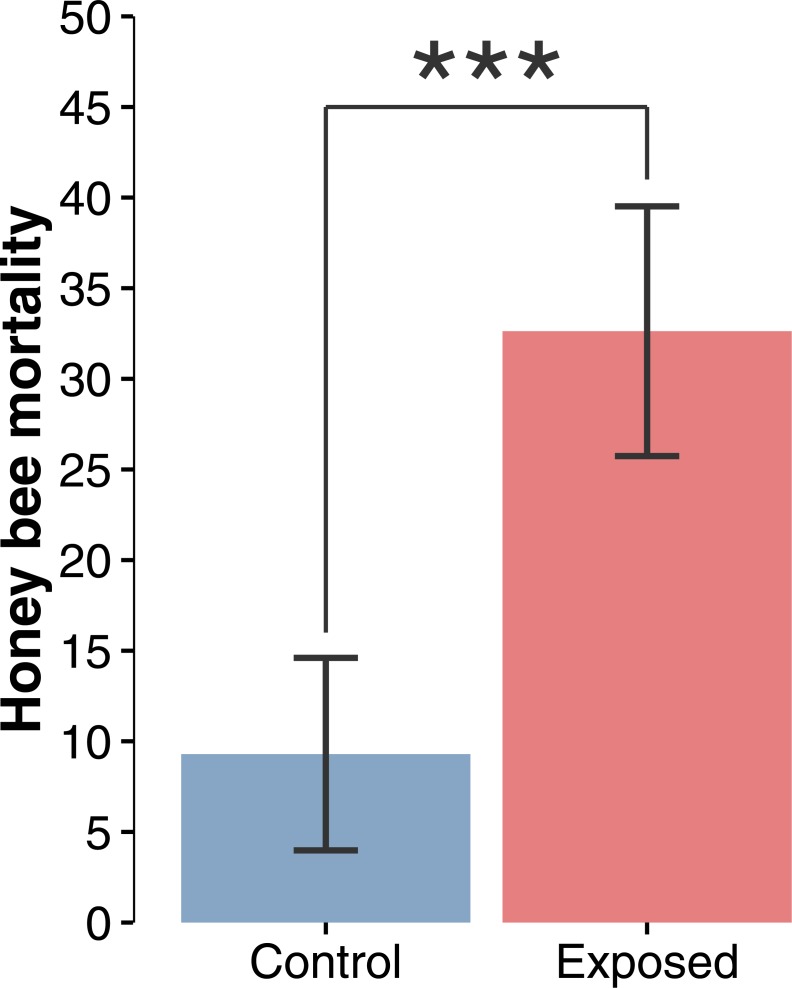
Honey bee mortality index. Mean (±CI) number of dead bees collected in front of hives during a 48-hour period over the whole corn planting season (*F*_1,22_ = 19.44; *p* = 0.0002).

## Results

### Honey bee mortality index

**Figure 3:**
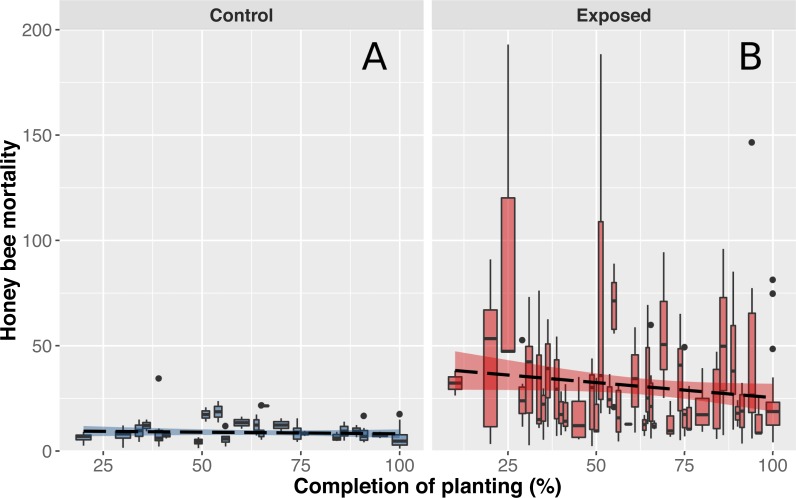
Honey bee mortality in function on planting completion. Mean (±CI) number of dead bees collected in front of hives from apiaries in corn-free environment (A) or near intensive corn cultivation (B) during a 48-hour period. Data shown on the *x*-axis indicate daily progression of corn planting (0% = planting has not begun, 100% = planting is over) to account for different corn planting duration of each year. Progression of corn planting was determined by estimates provided by agronomists from the government’s ministry of agriculture, provincial crop insurance reports and regional fertilizer sales (which immediately follow corn planting).

Compilation and statistical analysis of numbers of dead honey bees revealed a significant difference (*F*_1,22_ = 19.440897, *p* = 0.0002) in observed mortality during the corn planting period between apiaries from areas with intensive corn cultivation and those from corn-free environment, respectively 32.6 and 9.3 dead bees per colony per 48 h (Fig. 2). Daily mortality was on average 3.51 times higher for colonies located in the vicinity of corn fields planted with neonicotinoid-treated seeds. Mortality levels were not statistically different between sites or years within same treatment, and did not vary as a function of time (Figs. 3A and 3B) during the corn planting season.

### Multi-residue analyses of dead honey bees

**Figure 4:**
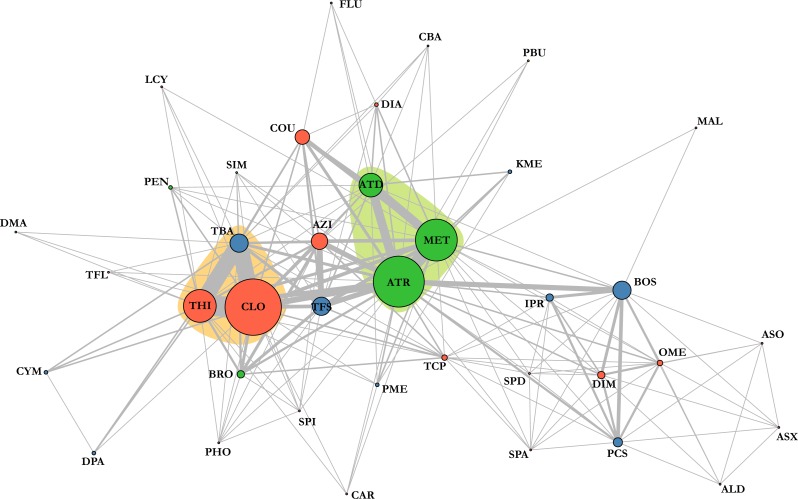
Pesticide detection network highlights the combinations of agrochemicals that were detected in a single sample. The wider the circle, the more frequent this compound was detected; the wider the line, the more frequent the combination was. Insecticides are marked in red, fungicides in blue, herbicides in green and synergist in yellow. Coloured areas indicate most common cocktails of chemicals. ALD, Aldicarb; ASO, Aldicarb sulfone; ASX, Aldicarb sulfoxide, ATR, Atrazine; AZI, Azinphos-methyl; BOS, Boscalid; BRO, Bromoxynil; CAR, Carbaryl; CBA, Carbendazim; CLO, Clothianidin; COU, Coumaphos; CYM, Cymoxanil; DAT, Desethyl atrazine; DIA, Diazinon; DIM, Dimethoate; DMA, Dimethenamid; DPA, Diphenylamine; FLU, Fludioxonil; IPR, Iprodione; KME, Kresoxym methyl; LCY, *λ* cyhalothrin; MAL, Malathion; MET, Metolachlor; OME, Omethoate; PBU, Piperonyl butoxide; PCS, Pyraclostrobin; PEN, Pendimethalin; PHO, Phosmet; PME, Pyrimethanil; SIM, Simazine; SPA, Spinetoram A; SPD, Spinetoram D; SPI, Spinosad A; TBA, Thiabendazole; TCP, Thiacloprid; TFL, Tau-fluvalinate; TFS, Trifloxystrobin; THI, Thiamethoxam.

**Figure 5:**
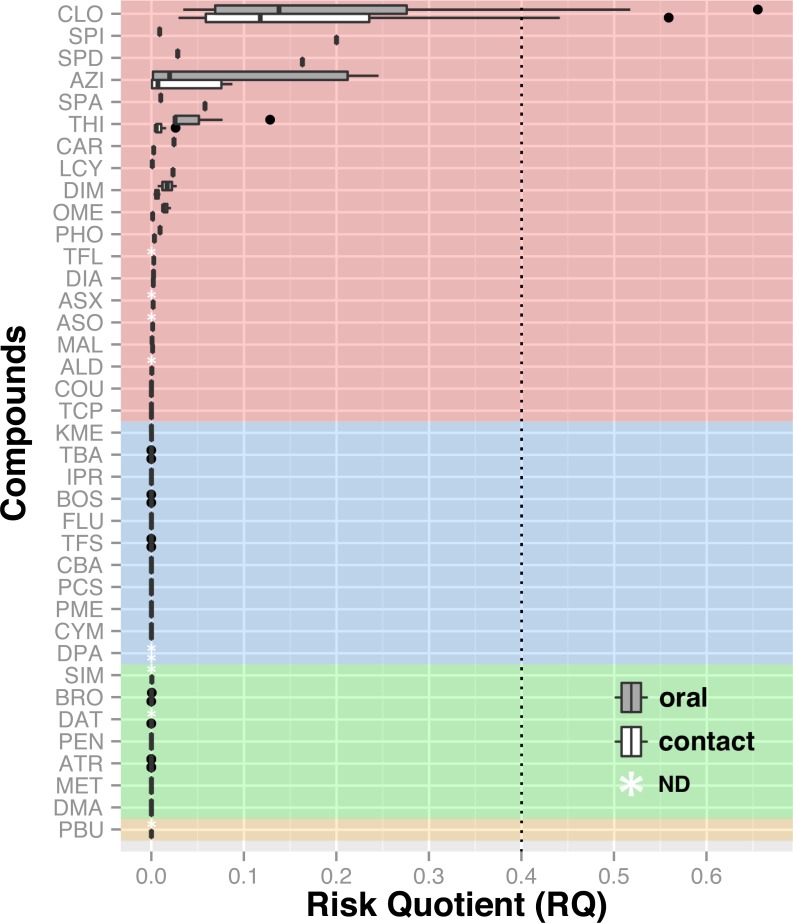
Risk assessment based on found concentrations in dead bees and known LD^50^. Insecticides are highlighted in red, fungicides in blue, herbicides in green and synergist in yellow. The acceptable limit of concern was set at 0.4 (RQ) based on the historic average dose response relationship for acute studies with bees as described in the White Paper (EFED, PMRA & CDPR, 2012). The neonicotinoid clothianidin is the only compound whose RQ exceeds the concern threshold. Hence, it poses the greatest toxicity risk to honey bees relative to other agrochemicals. ALD, Aldicarb; ASO, Aldicarb sulfone; ASX, Aldicarb sulfoxide; ATR, Atrazine; AZI, Azinphos-methyl, BOS, Boscalid; BRO, Bromoxynil; CAR, Carbaryl; CBA, Carbendazim; CLO, Clothianidin; COU, Coumaphos; CYM, Cymoxanil; DAT, Desethyl atrazine; DIA, Diazinon; DIM, Dimethoate; DMA, Dimethenamid; DPA, Diphenylamine; FLU, Fludioxonil; IPR, Iprodione; KME, Kresoxym methyl; LCY, *λ* cyhalothrin; MAL, Malathion; MET, Metolachlor, OME, Omethoate; PBU, Piperonyl butoxide; PCS , Pyraclostrobin; PEN, Pendimethalin; PHO, Phosmet; PME, Pyrimethanil; SIM, Simazine; SPA, Spinetoram A; SPD, Spinetoram D; SPI, Spinosad A; TBA, Thiabendazole; TCP, Thiacloprid; TFL, Tau-fluvalinate; TFS, Trifloxystrobin, THI, Thiamethoxam.

Chemical analyses of dead honey bees indicated that they were exposed to various agricultural chemicals throughout the corn planting period. A total of 38 different pesticides and metabolites, 19 insecticides, 11 fungicides, seven herbicides and one synergist, were found in the 74 dead honey bee samples (≥100 specimens) that were analysed throughout the study. In the 16 dead honey bee samples collected in control areas (apiaries in corn-free agricultural areas), three pesticides were identified (thiabendazole, clothianidin and thiamethoxam), with some samples containing two pesticides and an average of 1 chemical per sample (Table 1). Of these three pesticides, the fungicide thiabendazole was detected at levels above the limit of quantification, but levels of the neonicotinoid insecticides clothianidin and thiamethoxam were consistently below the limit of quantification, and therefore well below concentrations known to cause acute bee mortality (Lundin et al., 2015). Analysis of the 58 exposed samples collected from apiaries in neonicotinoid-treated corn intensive areas identified 32 pesticides and metabolites, with an average of 3.48 chemicals per sample and up to nine different compounds in a single sample (Table 2). Visualisation of pesticide detections in the form of a network highlights that the three most frequent pesticide co-occurence are clothianidin-thiamethoxam (unsurprisingly, as thiamethoxam is a known precursor of clothianidin), clothianidin-thiabendazole and thiamethoxam-thiabendazole and the most frequent triads of chemicals, which are clothianidin-thiamethoxam-thiabendazole in 30% of samples and atrazine-desethyl atrazine-metolachlor in 15% of samples (Fig. 4). Pesticide risk assessment based on the acute risk quotient (RQ) indicates that the vast majority of compounds score below the threshold level for concern (0.4) (EFED, PMRA & CDPR, 2012). RQ is expressed as the ratio of exposure estimate, in this case, concentrations of active ingredient found in dead bees, to point estimates of effects, as established by the acute contact and oral lethal dose to 50% of the organisms tested (LD50). For example, if clothianidin’s concentration in a given sample was of 19 µg/kg, its oral LD50 at 24 h is of 0.00368 µg/bee and considering a mean honey bee body weight of 0.128 g (EFED, PMRA & CDPR, 2012), then the corresponding RQ value would be of 0.66 ($19\times \left( 0.128\div 1,000 \right) \div 0.00368$). The neonicotinoid clothianidin was the only compound whose acute RQ value exceeded the acceptable limit and is therefore an identified source of concerns (Fig. 5).

**Table 1:** Summary of multi-residue analyses from control apiaries. Number of detections, concentrations and LD_50_ for all actives ingredients identified in samples (16) collected in front of colonies in a neonicotinoid-free agricultural environment during corn planting (LOQ = 1 µg/kg, LOD = 0.1 µg/kg).

Pesticide	Class^a^	Detection	(%)	Concentrations^b^ (µg/kg)				LD_50_ (ug/bee)	
				Min	Max	Mean	SEM	Contact	Oral
Thibendazole	FUNG	3	18.8	1.00	1.00	1	–	34^c^	4
Clothianidin	NEO, S	0	0.0	–	–	–	–	0.0439	0.00368
		(11 < LOQ)	(68.8)						
Thiamethoxam	NEO, S	0	0.0	–	–	–	–	0.024	0.005
		(2 < LOQ)							

**Notes.**

a Class: FUNG, fungicide, NEO, neonicotinoid, S, systemic.

b Concentrations (Min, Max, Mean and SEM) for detections >  LOQ.

c University of Hertfordshire (2013). The Pesticide Properties DataBase (PPDB) developed by the Agriculture & Environment Research Unit (AERU), University of Hertfordshire, 2006–2013 (Access: http://sitem.herts.ac.uk/aeru/ppdb/en/index.htm).

LD50 information from the Pesticide Ecotoxicity Database of the Office of Pesticide Programs, Ecological Fate and Effects Division, of the US Environmental Protection Agency, unless otherwise noted.

**Table 2:** Summary of multi-residue analyses from exposed apiaries. Number of detections, concentrations and LD_50_ for all actives ingredients identified in samples (*N* = 58) collected in front of colonies in a neonicotinoid dominated environment during corn planting (LOQ = 1 µg/kg, LOD = 0.1 µg/kg).

Pesticide	Class^a^	Detection	(%)	Concentrations^b^ (µg/kg)				LD_**50**_ (ug/bee)	
				Min	Max	Mean	SEM	Contact	Oral
Clothianidin	NEO, S	31	53.5	1.00	19	5.81	0.86	0.0439	0.00368
Atrazine	HERB, S	28	48.3	1.00	21.00	4.11	1.07	>97	100^a^
Metolachlor	HERB, PS	23	39.7	1.00	8.00	2.78	0.42	>110	>110
Thiamethoxam	NEO, S	18	31.0	1.00	5.00	1.61	0.26	0.024	0.005
		(9 < LOQ)	(15.5)						
Desethyl atrazine	HERB	13	22.4	1.00	33.00	6.69	2.32	>97	ND
Thiabendazole	FUNG	10	17.2	1.00	4.00	1.50	0.31	34^c^	4
		(15 < LOQ)	(25.9)						
Boscalid	FUNG, S	10	17.2	5.00	90.00	28.50	8.55	>200	>166
Trifloxystrobin	FUNG, PS	10	17.2	1.00	25.00	7.20	2.65	200	>200^c^
Azinphos-methyl	OP	9	15.5	1.00	287	98.89	43.62	0.42	0.15
Coumaphos	OP	8	13.8	1.00	3.00	1.63	0.32	20.29	2.99
Pyraclostrobin	FUNG	5	8.6	1.00	5.00	2.40	0.75	>100	>73.1^c^
Bromoxynil	HERB	4	6.9	3.00	7.00	4.50	0.87	14.5	5
Dimethoate	OP	4	6.9	3.00	12.00	7.50	1.94	0.16	0.056
Iprodione	FUNG	4	6.9	5.00	8.00	6.75	0.75	>120	>25^c^
Omethoate	OP	3	5.2	5.00	8.00	6.00	1.00	0.48	0,048^c^
Thiacloprid	NEO, S	3	5.2	1.00	7.00	3.33	1.86	37.83	17.32
Cymoxanil	FUNG	2	3.5	1.00	1.00	1.00	–	>25	>85.3^c^
Diazinon	OP	2	3.5	1.00	6.00	3.50	2.50	0.22	0.2
Diphenylamine	FUNG	2	3.5	2.00	6.00	4.00	2.00	ND	ND
Kresoxym methyl	FUNG	2	3.5	11.00	18.00	14.50	3.50	>25	>110^c^
Pendimethalin	HERB	2	3.5	3.00	3.00	3.00	–	49.8	>101.2^c^
Pyrimethanil	FUNG	2	3.5	1.00	2.00	1.50	0.50	100	100
Aldicarb	CARB, S	1	1.7	1.00	1.00	1.00	–	0.28	ND
Aldicarb sulfone	CARB, S, MET	1	1.7	3.00	3.00	3.00	–	0.28	ND
Aldicarb sulfoxide	CARB, S, MET	1	1.7	4.00	4.00	4.00	–	0.28	ND
Carbaryl	CARB, PS	1	1.7	21.00	21.00	21.00	–	1.1	0.11
Carbendazim	FUNG, S	1	1.7	3.00	3.00	3.00	–	>50	>756^c^
Dimethenamid	HERB	1	3.4	1.00	1.00	1.00	–	>98	>1,000
Fludioxonil	FUNG	1	1.7	14.00	14.00	14.00	–	>100^a^	>100
λ cyhalothrin	PYR	1	1.7	7.00	7.00	7.00	–	0.038	0.91^c^
Malathion	OP	1	1.7	2.00	2.00	2.00	–	0.2	0.38
Phosmet	OP	1	1.7	27.00	27.00	27.00	–	1.06	0.37
Piperonyl butoxide	SYN	1	1.7	1.00	1.00	1.00	–	>11	ND
Simazine	HERB, S	1	1.7	366.00	366.00	366.00	–	96.7	ND
Spinetoram A	SPI	1	1.7	11.00	11.00	11.00	–	0.024	0.14^c^
Spinetoram D	SPI	1	1.7	31.00	31.00	31.00	–	0.024	0.14^c^
Spinosad A	SPI	1	1.7	4.00	4.00	4.00	–	0.0029	0.057^d^
Tau-fluvalinate	PYR	1	1.7	4.00	4.00	4.00	–	0.2	ND

**Notes.**

a Class: CARB, carbamate; FUNG, fungicide; HERB, herbicide; MET, metabolite; OP, organophosphate; NEO, neonicotinoid; PS, partially systemic; PYR, pyrethrinoid; S, systemic; SPI, spinosine; SYN, synergist.

b Mean and SEM for detections >  LOQ.

c University of Hertfordshire (2013). The Pesticide Properties DataBase (PPDB) developed by the Agriculture & Environment Research Unit (AERU), University of Hertfordshire, 2006–2013 (Access: http://sitem.herts.ac.uk/aeru/ppdb/en/index.htm).

d Miles et al., 2011. Effects of spinosad on honey bees (*Apis mellifera*): findings from over ten years of testing and commercial use. 11th International Symposium of the ICP-BR Bee Protection Group. DOI: 10.5073/jka.2012.437.032. (Access: http://pub.jki.bund.de/index.php/JKA/article/viewFile/1958/2334)

e LD50 information from the Pesticide Ecotoxicity Database of the Office of Pesticide Programs, Ecological Fate and Effects Division, of the US Environmental Protection Agency, unless otherwise noted.

### Intracolonial population dynamic model

**Figure 6:**
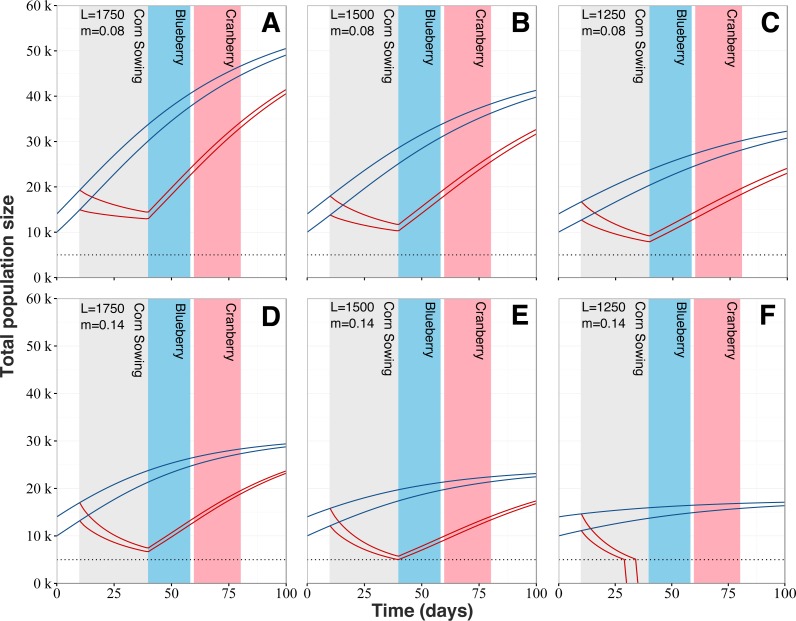
Honey bee population dynamics of six simulated colonies. Model is based on the mathematical models of Khoury, Myerscough & Barron (2011). Simulations start at 10,000 and 14,000 bees, which is the average springtime colony size for our study area. L corresponds to the queen’s daily egg-laying rate and varies from 1,750 to 1,250 to account for (A and D) fast, (B and E) regular and (C and F) slow colony growth. *m* is the normal level of forager mortality (either 0.08 or 0.14) which is multiplied by a factor of 3.51 for colonies in proximity to neonicotinoid seed-coated corn during planting period. Blue lines for colonies located in corn-free agricultural environment and red lines for colonies in vicinity neonicotinoid seed-coated corn fields. Collapse threshold is set at 5,000 bees (dotted line) based on the European Guidance Document on risk assessment on bees (EFSA, 2013).

Our model reveals that increased mortality levels linked with the planting of neonicotinoid-treated corn has the potential to strongly impact colony dynamics (Fig. 6). Regardless of the queens’ egg laying rate and typical mortality levels, populations from exposed colonies would follow a marked decline during the planting period of neonicotinoid-treated corn, after which colony growth would start anew. Simulations show that exposure to planting of neonicotinoid-treated corn by itself should not be sufficient to cause the collapse of exposed colonies. However, the worst-case scenario predicts colony collapse before the end of the corn planting period (Fig. 6F). Also, population sizes of exposed and surviving colonies are predicted to be considerably reduced at key moments during the Quebec’s beekeeping season, which are during the lowbush blueberry and cranberry crop pollination service periods. For instance, at the beginning of the lowbush blueberry pollination season beginning around early June, exposed colonies are predicted to be at best 2.33 times (Fig. 6A) and at worst 3.47 times (Fig. 6E) smaller than typical unexposed colonies. As for the cranberry pollination season, usually beginning in early July, colonies are still thought to be affected even if they partially recovered. According to our simulations, predicted populations of exposed colonies would still be 1.65 to 2.16 times smaller (respectively Figs. 6A and 6E) than expected populations of unexposed colonies. After the 100-days period, population sizes of exposed colonies are still predicted to be smaller than expected population of typical colonies by several thousand individuals, between 9,000 (Fig. 6A) to 5,600 (Fig. 6E) fewer bees.

## Discussion

This study indicates that springtime honey bee mortality is intensified when apiaries are located in the vicinity of neonicotinoid-coated corn fields, impairing colony development and bearing potential contribution to collapse. Our results also confirm that honey bees in Southern Quebec are regularly exposed to combinations of various pesticides, among which clothianidin was the most frequent occurrence and the only active ingredient to exceed threshold for concern. Furthermore, we highlight the special concern posed by co-occurrence of multiple pesticides, especially the clothianidin-thiamethoxam-thiabendazole triad, as it was the most frequent concurrent detection.

### Honey bee mortality index

Throughout the entire corn planting season, honey bee colonies established near neonicotinoid-treated fields showed a 3.51 times higher daily mortality index (Fig. 2). In temperate climates, this increased loss of foragers can be a cause for concern as spring is a particularly challenging time for colonies (Mattila & Otis, 2006; Betti, Wahl & Zamir, 2016). Resumption of intensive brood rearing in late winter and early spring may exhaust the colony’s pollen reserve before additional or sufficient resources become available in the environment (Mattila & Otis, 2006). Increased bee mortality would further impair the colony’s foraging efficiency and intensify pollen shortages. Should that happen, nutritional deficiencies would trigger a decline in brood production, which could even come to a complete halt (Mattila & Otis, 2006). Declining honey bee populations would result in seasonal vulnerability which would leave the colony more susceptible to other hazards (Betti, Wahl & Zamir, 2016).

As anticipated, mean mortality levels of control colonies were particularly stable and did not differ throughout the corn planting season (Fig. 3A). However, contrary to expectations, worker mortality of exposed colonies did not significantly differ as a function of time (Fig. 3B). We initially believed that mortality levels would be higher at the beginning of corn planting and gradually subside during spring as a similar pattern was observed with acetylcholinesterase expression in bumblebees (Samson-Robert et al., 2015). Our results did suggest that mean mortality levels tended to decrease over the season but the magnitude of variability in mortality between days and colonies of exposed sites did not allow for the detection of a significant effect (Fig. 3B). We hypothesize that weather conditions are a key driver of pesticide related bee deaths which would explain the variability observed in mortality levels between days. Since honey bees do not venture outside when it is raining and corn cannot be planted when heavy or frequent precipitation occurs, both pesticides exposure and honey bee deaths should be minimal. When weather conditions become favourable again, corn planting and honey bee foraging resume and mortality should consequently rise. We also hypothesize that proximity of on-going plantings with honey bee hive and bees foraging areas, rather than regional progression of plantings, play a key role in pesticide exposure and honey bee deaths, which would explain the observed variability in mortality levels between colonies of exposed sites.

### Multi-residue analyses of dead honey bees

Our results also indicate that honey bees from bee yards established in the vicinity of agricultural fields are not only widely exposed to neonicotinoid insecticides during corn planting, but also to a wide array of agricultural pesticides that play an important role in impairing colony health (Tables 1 and 2). Pesticide residue concentrations in dead bees were generally low and seldom exceeded LD_50_. However, many pesticides are rapidly metabolized in live bees, which may explain the general lack of high residue concentrations in recently dead bees. Nicotinic insecticides are particularly problematic in that regard since they are known to be metabolized within a few hours (Suchail, Debrauwer & Belzunces, 2004; Suchail et al., 2004; Cresswell et al., 2014; Blacquière & Van der Steen, 2017), making their detection especially tricky. Moreover, metabolites of neonicotinoids, most of which were not targeted by the chemical analyses, have also been shown to be highly toxic for bees (Suchail, Guez & Belzunces, 2001; Nauen et al., 2003; Blacquière et al., 2012) increasing the long-term toxicity of these compounds. Most pesticide detections also revealed the co-occurrence of many active ingredients within the same samples (Fig. 4), which would be expected to exhibit enhanced toxicity if they share the same mode of action or target site (Yu, 2014; Zhu et al., 2014) or if compounds compete for a limited pool of detoxification enzyme (Berenbaum & Johnson, 2015; Johnson, 2015). Neonicotinoids thus pose a particular threat, as they are known to be additively toxic when they occur together (Andersch, Jeschke & Thielert, 2010; Pavlaki et al., 2011; Palmer et al., 2013), this was the case for 31% of our samples (Fig. 4). Neonicotinoids are also known to interact additively with various other agrochemicals. As such, combined neonicotinoid exposure to certain pyrethroids, like *λ*-cyhalothrin, has been related to increased toxicity (Gill, Ramos-Rodriguez & Raine, 2012; Chen et al., 2015). Combined exposure with fungicides like boscalid (Tsvetkov et al., 2017), trifloxystrobin (Wachendorff-Neumann et al., 2012) and ergosterol inhibitor (Iwasa et al., 2004; Thompson et al., 2014; Johnson, 2015; Sgolastra et al., 2017) also result in synergistic toxicity. In the last six years, 12 patent applications have also claimed or demonstrated additive toxicity between the fungicide thiabendazole and thiamethoxam (Donley, 2016), which occurred together in 30% of our samples. Herbicidal compounds have also been shown to be additively toxic when occurring together with neonicotinoids, as was the case with atrazine in 19% of our samples. Finally, combined exposure with in-hive acaricides, such as coumaphos, results in enhanced toxicity in addition with impaired memory and olfactory learning (Williamson & Wright, 2013; Berenbaum & Johnson, 2015; Johnson, 2015). Of all the insecticides identified in dead honey bee bodies, those of the neonicotinoid family, clothianidin and thiamethoxam, were detected far more frequently than others. Furthermore, clothianidin was the only pesticide whose acute RQ exceeded the threshold for concern (Fig. 5) and thereby poses the highest risk for honey bees, which is in accordance with the findings of Sánchez-Bayo & Goka (2014). Taking into account (1) the known high toxicity of nitroguanidine neonicotinoid insecticides (Goulson, 2013); (2) concentration levels found in dead bees in our study; (3) the possible synergistic interactions with other agrochemicals identified in collected dead bees; and (4) the synchronism between the timing of honey bee deaths and concurrence with planting of treated corn in the vicinity, our study concludes that exposure to neonicotinoid compounds, mostly clothianidin, was the most likely cause of the bee mortality observed at the hives during our study.

### Impaired colony growth and contributions to collapse

Unusually high spring forager mortality (Fig. 2) might represent a heavy burden for colonies, assuredly rendering them more vulnerable to other threats and potentially causing their collapse. When implementing the increased in-field mortality into a honey bee population dynamic model, all scenarios exhibited a major deviation from the expected colony dynamic (Fig. 6). When considering the 100-day period, all exposed colonies suffered an important developmental setback, which has the potential to, in extreme scenarios (Fig. 6F), cause the collapse of the colonies. In most cases however, simulations show that colonies should survive the increased mortality and recover over the course of the season, given the proper care and favourable conditions. Nonetheless, colonies seldom encounter such ideal conditions and in reality must usually deal with several pathogens, parasites and predators (Potts et al., 2010; Vanbergen et al., 2013; Goulson et al., 2015; Gill et al., 2016; Klein et al., 2017). In bad-case scenarios (Figs. 6D and 6E ), when the corn planting period ends, colonies are expected to dwindle just above 5,000 individuals, which represents the lowest viable colony size (Henry et al., 2012; EFSA, 2013). With such small populations, even a slight disruption of the colony, caused by any type of stress (dietary, parasites, pathogens, weather, transhumance, etc.), might be sufficient to cause its collapse. Recently, Henry et al. (2015) confirmed that field exposure to neonicotinoid was indeed associated with increased forager mortality. They also concluded that colonies compensate for the excess mortality without impairing colony growth or honey production. These results appear to be in contradiction with our own conclusions. However, Henry et al. colonies were exposed to oilseed rape grown from seeds treated with neonicotinoid and research on honey bees have repeatedly shown little adverse effects at colony-level in this particular crop (Cutler & Scott-Dupree, 2007; Pohorecka et al., 2012; Pilling et al., 2013; Cutler & Scott-dupree, 2014). Being particularly attractive to honey bees, it seems counter intuitive that this crop would not adversely impact colonies. However, oilseed rape produces abundant sugar-rich nectar (Kevan, Lee & Shuel, 1991; Pernal & Currie, 1997) and good quality pollen (Somerville, 2001) allowing for fast and sustained colony development. We believe these benefits outweigh the costs of exposure to neonicotinoid residues from treated seeds.

### Pollination services

A recent study by Stanley et al. (2015) showed, for the first time, that exposure to neonicotinoid pesticides induced colony-level reduction in pollination services provided by bumblebees to apples. In the case of honey bees, impaired colony development as shown in our population dynamic models may negatively impact both pollination services and honey production, resulting in important economic losses for beekeepers and berry growers in Quebec. For one, to be economically profitable for beekeepers, or even considered for pollination services, honey bee colonies need to meet population size standards in Quebec (FAQ, 2015). Immediately following chronic exposure from corn plantings, predicted honey bee populations of bad-case scenarios (Figs. 6D and 6E) would not meet the minimal required standard of 7,000 bees and could simply not be rented for pollination services in Quebec. Even mid-summer blooming crops would potentially deal with the after effects of this chronic exposure, as predicted population of colonies would still only be a fraction of the required strength. One common way of dealing with weak colonies is to combine them together so the resulting colonies, although fewer in numbers, would then have the required strength for commercial pollination. As chronic exposure to neonicotinoid compounds reduces the number of honey bee colonies available for rent, pollinator-dependent crops might suffer from this pollination service shortage. For example, lowbush blueberry and cranberry crops are two of Quebec’s fastest growing productions and annually monopolise 90% of the colonies on rent throughout the province (ISQ, 2014). Over the last 10 years, honey bee colony rental prices for crop pollination has increased by more than 60% due to the increasing pollination demand from these two crops (Belzile & Li, 2014; ISQ, 2014). Chronic exposures to toxic chemicals thus forces beekeepers to deal with weakened colonies, more vulnerable to parasites and pathogens, worth less for pollination services, sometimes not rentable at all and in all cases producing less honey. Crop growers also bear the consequences as weaker bee colonies are not as efficient at pollinating their crops and rental prices are likely to increase if supply of bee hives becomes a negative issue.

## Conclusions

Extensive spring planting of neonicotinoid-coated corn resulted in increased daily mortality of individuals from commercial honey bee colonies located in the vicinity (<500 m). Neonicotinoid compounds were the prevailing insecticides detected in dead honey bees from our study with clothianidin being the only compound whose acute RQ exceeded the threshold for concern. The consequence of this type of agrochemical contamination strongly limits honey bee colony development, potentially affecting the number of available foragers, and reducing their colony effectiveness for crop pollination and consequently their monetary value for hive rental. These adverse conditions can likely lead to colony collapse. Additionally, the widespread use of neonicotinoid treated-corn has been repeatedly questioned. Studies from Europe and North America alike have shown that prophylactic use bears insignificant to little increase in yield and that more sustainable pest control alternatives are available (Quesnel, 2005; Goulson, 2013; Furlan & Kreutzweiser, 2014; Labrie et al., 2014; Krupke et al., 2017). Our ISQ provides further evidence of the key role of neonicotinoids in the declining health of honey bee colonies and highlights the potential effects of bee exposure to common combinations of agrochemicals. Policy makers should prioritize the need for future research into pesticide interactions.

##  Supplemental Information

10.7717/peerj.3670/supp-1 Supplemental Information 1 Honeybee mortality count data set The file contains the following data columns: date (Date at which samples were collected); year (2012, 2013); Treatment (Exposed, Control); site (Individual site name, E1, E2, E3, E4, E5, E6, E7, E8, E9, E10, E11, E12, E13, E14, C1, C2, C3, C4, C5, C6, C7, C8); colony# (Individual colony identification number); count (Number of honeybee bodies on a one square meter piece of cloth); NA is used to indicate missing data.

10.7717/peerj.3670/supp-2 Supplemental Information 2 Honeybee pesticide analyses (LC-MSMS) data set The file contains the following columns: date (Date at which samples were collected); year (2012, 2013); site (Individual site name, E2, E3, E4, E5, E6, E8, E9, E12, E13, E14, C1, C4, C5, C6, C8); Treatment (Exposed, Control); all following columns refer to active ingredients (Concentration, in ppb). NA is used to indicate missing data.

10.7717/peerj.3670/supp-3 Supplemental Information 3 Honeybee population dynamic model data set The file contains the following data columns: laying_rate (Egg laying rate of queens as reported in scientific literature); mortality_rate (mortality rate as reported in scientific literature); days (number of days since the beginning of simulations, from 0 to 100); pop_size (Initial size of simulated populations); treatment (Treatment of colonies: “Con” for no exposure, “Exp” for regular 30 day exposure, “10,000” for colony population beginning at 10,000 individuals and “14,000” for colony population beginning at 14,000 individuals); trt (Unique letter referring to each individual treatment: A, B, C, D). There is no missing data.
